# Antimicrobial copper alloy surfaces are effective against vegetative but not sporulated cells of gram-positive *Bacillus subtilis*

**DOI:** 10.1002/mbo3.276

**Published:** 2015-07-16

**Authors:** Kaungmyat San, Janet Long, Corinne A Michels, Nidhi Gadura

**Affiliations:** 1 Department of Biological Sciences and Geology, Queensborough Community College – CUNY222-05 56th Avenue, Bayside, New York, 11364; 2 Biology Department, Queens College – CUNY65-30 Kissena Boulevard, Flushing, New York, 11367

**Keywords:** Antimicrobial copper, lipid peroxidation, mechanism copper surface killing

## Abstract

This study explores the role of membrane phospholipid peroxidation in the copper alloy mediated contact killing of *Bacillus subtilis*, a spore-forming gram-positive bacterial species. We found that *B. subtilis* endospores exhibited significant resistance to copper alloy surface killing but vegetative cells were highly sensitive to copper surface exposure. Cell death and lipid peroxidation occurred in *B. subtilis* upon copper alloy surface exposure. In a sporulation-defective strain carrying a deletion of almost the entire *SpoIIA* operon, lipid peroxidation directly correlated with cell death. Moreover, killing and lipid peroxidation initiated immediately and at a constant rate upon exposure to the copper surface without the delay observed previously in *E. coli*. These findings support the hypothesis that membrane lipid peroxidation is the initiating event causing copper surface induced cell death of *B. subtilis* vegetative cells. The findings suggest that the observed differences in the kinetics of copper-induced killing compared to *E. coli* result from differences in cell envelop structure. As demonstrated in *E. coli*, DNA degradation was shown to be a secondary effect of copper exposure in a *B. subtilis* sporulation-defective strain.

## Introduction

Copper alloy surfaces rapidly kill a significant variety of microorganisms in both the laboratory and clinical setting (reviewed in Grass et al. 2011; Lemire et al. 2013). This antimicrobial activity is surprisingly broad ranging and targets gram-positive and gram-negative bacteria (Wilks et al. 2005, 2006; Noyce et al. 2006; Mehtar et al. 2008; Weaver et al. 2008, 2010; Warnes et al. 2010, 2012; Espírito Santo et al. 2011, 2012; Ibrahim et al. 2011; Warnes and Keevil 2011; Tian et al. 2012; Zhu et al. 2012; Bleichert et al. 2014; Cui et al. 2014), mammalian and bacterial viruses (Noyce et al. 2007; Li and Dennehy 2011; Bleichert et al. 2014; Warnes et al. 2015), and yeast (Ohsumi et al. 1988; Quaranta et al. 2011). In the clinical setting, the inclusion of copper alloy surfaces in a medical ICU room effectively reduced microbial bioload by over 83% and, most importantly, decreased the rate of hospital-acquired infections in these rooms by 58% (Grass et al. 2011; Schmidt et al. 2012; Lemire et al. 2013; Salgado et al. 2013). In view of the potential of copper alloy surfaces to control the spread of infection in venues such as hospitals, schools, and public transportation, it becomes essential to understand the mechanism by which copper kills. Knowing the mechanism of killing informs us about the possibility of developing strains resistant to metallic copper alloy surface exposure, an important consideration when selecting any antimicrobial agent.

A variety of gene functions have been identified in bacterial species that are responsible for maintaining proper intracellular and periplasmic levels of copper ions (reviewed in Brown et al. 1992; Cooksey 1993; Cervantes and Gutierrez-Corona 1994; Rademacher and Masepohl 2012; Bondarczuk and Piotrowska-Seget 2013; Guo et al. 2015). Copper resistance mechanisms include reduced copper import, enhanced expression of copper efflux systems, and enhanced expression of cell components that form copper complexes. Espírito Santo et al. (2008) investigated the contribution of a number of the copper resistance systems to survival of *E. coli* on copper surfaces. They found that mutant strains lacking the copper detoxification systems *CopA*, *Cus*, and *CueO* exhibited a slight increase in sensitivity to copper surface exposure. Conversely, a copper-resistant strain harboring the plasmid-borne *Pco* operon survived only 3-times longer than the parental host on a copper alloy surface but even this strain succumbed to dry copper surface killing in a few minutes. Espírito Santo et al. (2008) estimated that the amount of copper ion released during these few minutes of exposure was orders of magnitude less than the copper ion concentrations to which these strains are resistant when exposed in solution. It appears that the copper ion-resistant systems described above contribute to copper alloy surface-mediated killing but other mechanisms are of greater importance.

Several lines of evidence support the conclusion that surface-released-free copper ions are the causative agent in copper-mediated contact killing but may not be acting directly (Espírito Santo et al. 2008, 2010, 2011; Molteni et al. 2010; Elguindi et al. 2011; Zeiger et al. 2014). It has been proposed that free copper ions catalyze the nonenzymatic Fenton reaction at the site of bacterial contact and produce reactive oxygen species (ROS) that are the toxic agent responsible for microbial cell death. Studies by Espírito Santo et al. (2011) and others (reviewed in Grass et al. 2011) point to a component of the bacterial cell membrane as the primary target of copper alloy surface exposure. This is consistent with the limited impact of the various copper resistance systems to survival on copper alloy surfaces discussed above (Espírito Santo et al. 2008). Given the diversity of microorganisms sensitive to copper-mediated contact killing and the inability to isolate resistant strains, Hong et al. (2012) hypothesized that this membrane target is likely to be an essential component common to the membranes of all of these organisms. Furthermore, they proposed that the unsaturated fatty acids that are required to provide the essential fluidity of the phospholipid bilayer are a likely target. Working with the gram-negative bacteria *E. coli*, their results provide evidence that peroxidation of membrane phospholipids results in the loss of membrane competence that, in turn, stimulates a catastrophic cell death. Notably, Hong et al. (2012) showed that lipid peroxidation and cell death occurred prior to genomic DNA degradation and that cell death could occur even in the absence of genomic DNA degradation, clearly indicating that the latter is a secondary step in the cell death process.

We undertook the investigation reported here to explore the impact of copper alloy surfaces on *Bacillus subtilis*, an endospore-forming gram-positive bacterium. The study focused on the relationships among cell death, lipid peroxidation, and DNA degradation with the goal of broadening our hypothesis of the role of lipid peroxidation as the primary target of copper surface-mediated killing. Our findings suggest that the mechanism of killing in *B. subtilis* is similar to that in gram-negative *E. coli* but that the impact of copper alloy surface exposure in *B. subtilis* affected only vegetative cells. Killing and lipid peroxidation initiated immediately upon exposure to the copper surface without the delay observed in *E. coli* that produced a biphasic killing curve in this species (Hong et al. 2012). We propose that the mechanism of copper alloy surface killing in *B. subtilis* vegetative cells is similar to that found in *E. coli* but that the lack of this delay results from differences in the bacterial envelope structure between these gram-negative and gram-positive organisms.

## Materials and Methods

### Strains and growth conditions

The *Bacillus subtilis* strains used in this study were obtained from Dr. Patrick Piggot, Temple University, Philadelphia, PA. *B. subtilis* 168 strain BR151 (*trpC2 metB10 lys-3*) is the Spo^+^ parental strain from which the Spo^−^ mutant strain SL10950 was derived. Strain SL10950 contains a partial deletion of the *SpoIIA* operon designated *spoIIAB-ACΔ::neo* that removes the 3′ region of *spoIIAB* and the complete *spoIIAC* gene and replaces this with a cassette carrying the neomycin resistance gene (*neo*) thereby producing a sporulation negative (Spo^−^) and neomycin-resistant strain (Chary et al. 2005).

For liquid culture, the *B. subtilis* strains were grown in modified Schaeffer's sporulation medium (MSSM) that was supplemented with 3.5 *μ*g/mL neomycin (final concentration) for strain SL10950 (Schaeffer et al. 1965; Piggot and Curtis 1987). Schaeffer's Sporulation Agar supplemented with 3.5 *μ*g/mL neomycin as appropriate was used to titer the number of colony-forming units (CFU).

### Metal coupon cleaning protocol

Metal coupons consisting of a 1-inch^2^ sheet of a specified alloy composition were provided by the Copper Development Association, New York. The alloys used here are: C11000 (99.90% copper [wt/wt], C28000 (60% copper, 40% zinc [wt/wt], and 304 stainless steel (18% chromium, 8% nickel, 74% iron [wt/wt]). The coupons were degreased, cleaned, and sterilized as described in (Hong et al. 2012). The cleaned coupons were individually flame sterilized with 95% ethanol stored in sterile Petri dishes.

### Killing curves

*Bacillus subtilis* strains were grown at 37°C with aeration to early log phase (OD_600_ about 0.3) in MSSM media. Cells were harvested from 100 mL of culture, washed once with 0.85% NaCl, and resuspended in 0.85% NaCl to give a final volume 500 *μ*L. Using a sterile micropipette, 100 *μ*L samples of concentrated cells (approximately 10^10^ cells) were spread on the coupon surface in a sterile Petri dish and allowed to air dry, when the time course allowed. Drying was complete in about 15 min. Exposures times of <15 min are thus to be considered moist exposure. At the indicated time, cells were collected from an individual coupon surface by repeated washing and scraping with the micropipette tip with 100 *μ*L of sterile 0.85% NaCl. Survival was determined by dilution and plating on SSA media with 3.5 *μ*g/mL neomycin, as appropriate. The remainder of the sample was used for the TBARS assay or as a source of total genomic DNA, according to the methods described below. Each experiment was repeated at least three times.

### TBARS assay

The TBARS (Thiobarbituric Acid Reactive Substances) assay is described in detail at (Rael et al. 2004). Cells were recovered from the coupon by repeated washing with 100 *μ*L of sterile 0.85% NaCl and using the micropipette tip to scrape the surface. The entire sample was used for the TBARS assay, which was performed as described by the manufacturer (ZeptoMetrix Corp., Buffalo, NY, USA) as adapted by Hong et al. (2012). The absorbance at 532 nm of the reaction supernatant was determined using a Shimadzu Biospec-mini spectrophotometer. The amount of malondialdehyde (MDA) was determined by comparison to an MDA standard curve and is reported as nmoles per 10^9^ cells. Each experiment was repeated with three independent cultures.

### Genomic DNA extraction and gel electrophoresis

The Wizard Genomic DNA purification kit from (Promega Corporation, Madison, WI, USA) was used to isolate total genomic DNA. Cells were recovered from the coupon surface wash by centrifugation and the entire sample was used for the preparation of total genomic DNA, as described by the manufacturer and described in Hong et al. (2012). The extracted DNA was size separated by PAGE using a 1% agarose gel (0.5 g of agarose/50 mL TAE buffer) and visualized with ethidium bromide. It is important to note that this method extracts DNA only from vegetative cells and is not able to lyse the *B. subtilis* spores.

### Microscopic cell viability assay

The Invitrogen LIVE/DEAD® (Invitrogen LifeTechnologies, Grand Island, NY, USA) BacLight Bacterial Viability Kit (Cat. L7012) for microscopy and quantitative assays was used to visually monitor cell viability. Cells were stained as described in the manufacturer's protocol and observed using a Zeiss fluorescent Axioscope (Carl Zeiss Corp., Thornwood, NY, USA) (GFP and Rhodamine filters) and an AxoCam ICm1 camera. Total magnification used was 1000× oil immersion.

## Results

### Effects of exposure of *B. subtilis* endospore-forming and sporulation-defective strains to copper alloy surfaces

Metal surface exposure was achieved using 1-inch^2^ sheets of the indicated alloy, referred to as coupons (Hong et al. 2012). Two copper alloys were used in this study: alloy C11000 consisting of 99.90% copper (sometimes referred to as “pure” copper) and alloy C28000 consisting of 60% copper and 40% zinc. Stainless steel (S30400) was used as control. *B. subtilis* strain BR151 was grown in MSSM media, harvested by centrifugation, and resuspended in 0.85% NaCl. Approximately 10^10^ cells were spread evenly over the surface of each coupon under sterile conditions and allowed to air dry (approximately 15 min). At the indicated times, the bacterial cells were recovered from a coupon by vigorous washing and scraping. A small volume of the recovered sample was used to determine cell survival and the remainder used to assay lipid peroxidation by the TBARS assay.

**Figure 1:**
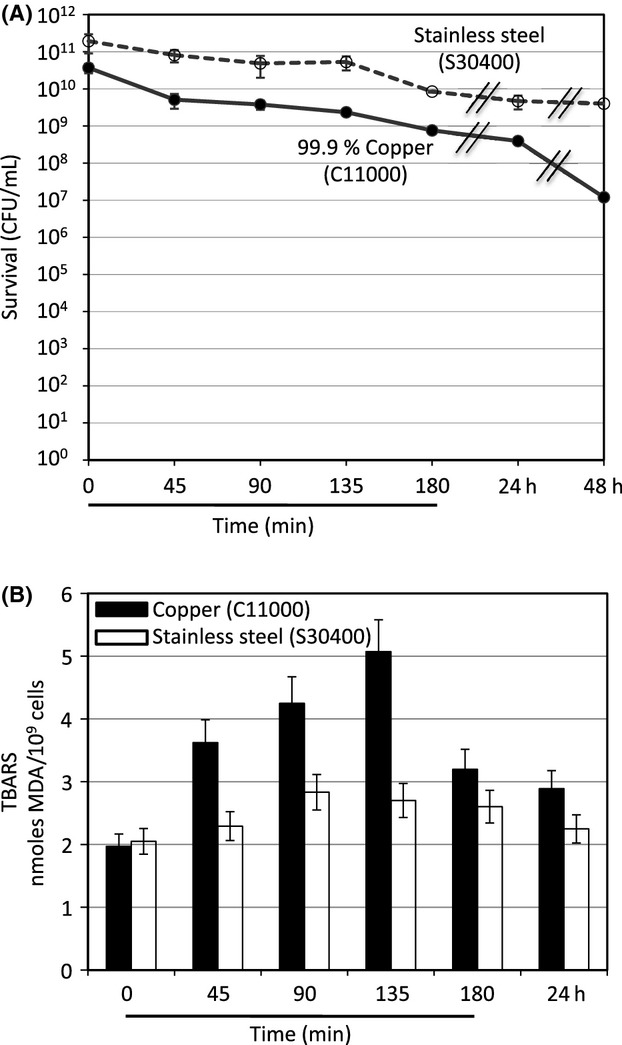
*Bacillus subtilis* survival and lipid peroxidation following exposure to metallic copper surface. *B. subtilis* strain BR151 was grown at 37°C with aeration to early log phase (OD_600_ about 0.3) in modified Schaeffer's sporulation medium. Cells were harvested by centrifugation from 100 mL of culture, and resuspended in 0.85% NaCl to a final volume of 500 *μ*L. 100 *μ*L of concentrated cells were spread over the surface of metal coupons of 304 stainless steel (S30400) or 99.90% copper (C11000). Panel A: At the indicated time of exposure, cells were washed from the coupon surface with 100 *μ*L of 0.85% NaCl, and samples of the cell suspension taken to titer survival. Panel B: Thiobarbituric Acid Reactive Substances (TBARS) assay was carried out on the remainder of the cell sample exposed and recovered from the coupon surface at the indicated times. The error bars indicate standard deviation from three independent cultures assayed in duplicate.

Figure1A compares survival on copper to that on stainless steel. At 45 min, survival on the copper coupon dropped to about 14%, significantly less survival than the 42% observed on the 304 stainless steel coupons. Over the following hours, a slow and steady decline in the number of survivors was observed on both copper and stainless steel until, at 24 h of exposure, survival began to decrease rapidly on the copper coupons but not on the stainless steel coupons.

Lipid peroxidation in *B. subtilis* strain BR151 cells exposed to these same copper and stainless steel coupons is shown in Figure1B. A significant increase in the level of lipid peroxidation was observed in cells exposed to copper compared to the stainless steel control but the kinetics differed somewhat from the kinetics of killing. Peroxidation initiated immediately at a high rate but the level peaked at 135 min before dropping to a lower level by 180 min of exposure. The level continued to decrease slowly over the next 24 h but remained higher than at time zero. The reason for the peak and subsequent decrease is not clear. It should be noted that the TBARS assay monitors the formation of malondialdehyde (MDA), a by-product of lipid peroxide breakdown, which might also be unstable under these experimental conditions and thereby produce the observed peak.

The results reported here differ significantly from our results with *E. coli* (Hong et al. 2012), most particularly with respect to the extent of copper alloy dependent killing. Hong et al. (2012) found about 9 logs of killing with few, if any, survivors after 45 min exposure to the C11000 copper alloy surface. Here we found only about 1 log of killing over the same time period. On the basis of studies of the endospore-forming bacterial species *Bacillus anthracis* (Bleichert et al. 2014) and *Clostridium difficile* (Weaver et al. 2008), we hypothesized that the apparent resistance to copper alloy surface killing might be a function of the endospore-forming ability of *B. subtilis* strain B151 (Piggot and Hilbert 2004). To explore this further, we determined survival and lipid peroxidation in the sporulation-defective strain SL10950 (Chary et al. 2005). Strain SL10950 is isogenic to BR151 but carries a deletion of almost the entire *SpoIIA* operon (*spoIIAB-ACΔ::neo*). Observation of both strains using bright field microscopy confirmed the absence of endospores in strain SL10950 under the growth conditions of this study (data not shown).

**Figure 2:**
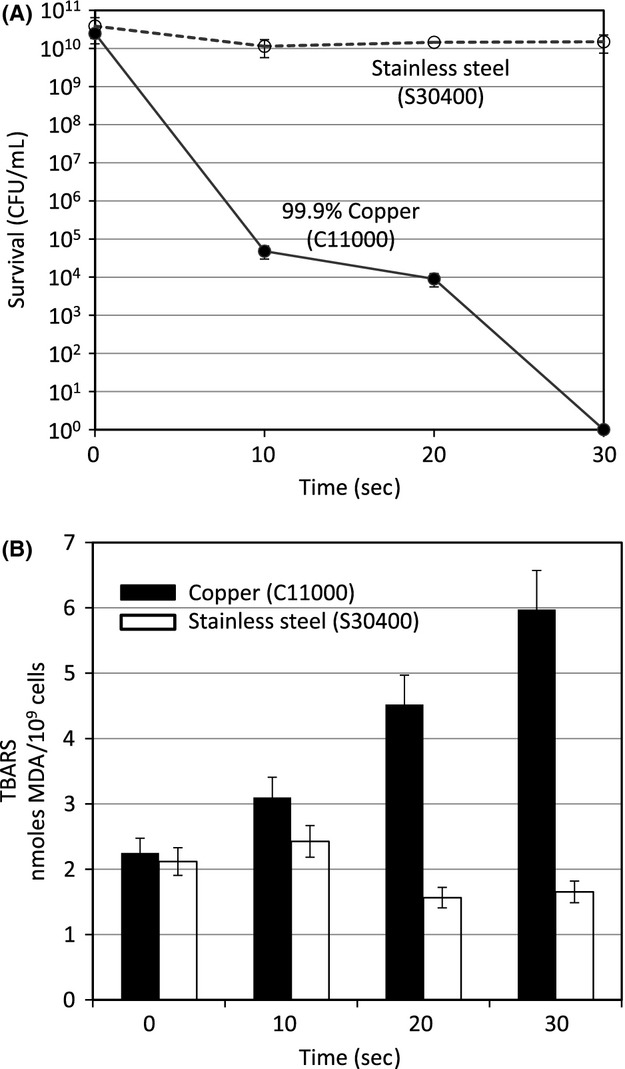
Survival and lipid peroxidation of a sporulation negative *Bacillus subtilis* strain following exposure to 99.90% copper alloy (C11000) surface. *B. subtilis* strain SL10950 (*spoIIAB-ACΔ::neo*) was grown at 37°C with aeration to early log phase (OD_600_ about 0.3) in modified Schaeffer's sporulation medium supplemented with 3.5 *μ*g/mL neomycin. Cells were harvested, exposed to metal coupons of 304 stainless steel (S30400) or 99.90% copper (C11000), washed from the coupon surface at the indicated time, and samples of the wash suspension taken to titer survival (Panel A) or for the Thiobarbituric Acid Reactive Substances (TBARS) assay (Panel B), as described in Figure1. Please note that the time course is in seconds of exposure. The error bars indicate standard deviation from three independent cultures assayed in duplicate.

Using the same procedures described for strain BR151 in Figure1, strain SL10950 was exposed to copper alloy C11000 and survival and lipid peroxidation followed (see Fig.2). Please note that the *Y*-axis in Figure2 is time in seconds in both panels A and B. Because of the extremely short exposure times, the cell suspension did not dry onto the coupon surface and thus this experiment should be considered “moist” copper alloy surface exposure. Amazingly, killing was essentially complete (over 10 logs reduction in the number of CFU) in 30 sec with no evidence of the initial delay observed with *E. coli* (Hong et al. 2012). Lipid peroxidation also initiated immediately and increased rapidly over the entire 30 sec.

**Figure 3:**
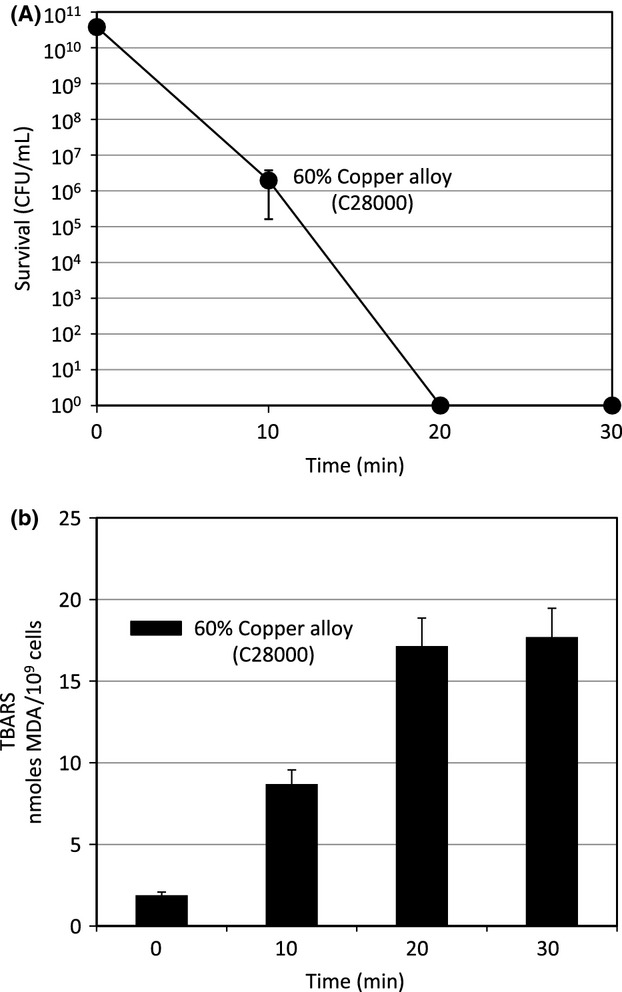
Survival and lipid peroxidation of a sporulation negative *Bacillus subtilis* strain following exposure to 60% copper alloy (C28000) surface. *B. subtilis* strain SL10950 (*spoIIAB-ACΔ::neo*) was grown in modified Schaeffer's sporulation medium supplemented with 3.5 *μ*g/mL neomycin as described in Figure2. Cells were harvested, exposed to coupons of 60% copper – 40% zinc alloy (C28000), washed from the coupon surface at the indicated time, and samples of the wash suspension taken to titer survival (Panel A) or for the Thiobarbituric Acid Reactive Substances (TBARS) assay (Panel B) as described in Figure1. Please note that the time course is in minutes of exposure. The error bars indicate standard deviation from three independent cultures assayed in duplicate.

In an effort to improve resolution, the experiment was repeated using the 60% copper – 40% zinc alloy C28000. As can be seen in Figure3, the rate of killing is slowed significant (measured in minutes) allowing survival and lipid peroxidation to be followed over a more manageable 30-min time course. A 20-min exposure to C28000 produced greater than 10 logs of killing and few if any survivors were found by 20 min. Lipid peroxidation initiated immediately and reached a plateau at the 20- and 30-min time points. The killing curve inversely mirrors lipid peroxidation clearly suggesting that the killing of *B. subtilis* is closely linked to copper-dependent lipid peroxidation.

### Genomic DNA degradation in spore-forming and sporulation-defective *B. subtilis* strains following exposure to copper alloy C11000 (99.9%) surfaces

**Figure 4:**
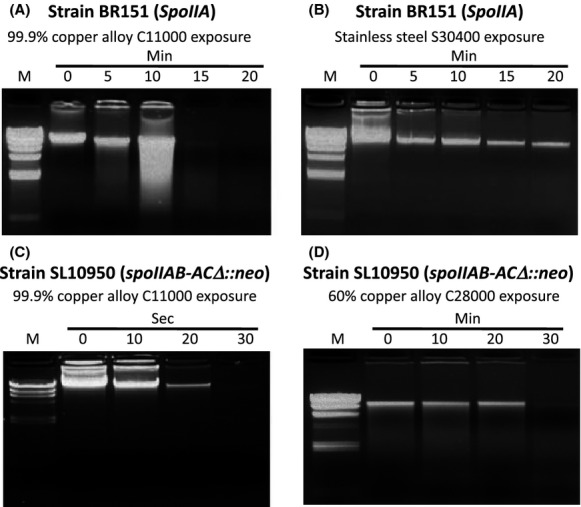
DNA degradation of *Bacillus subtilis* strains BR151 and SL10950 following exposure to 99.90% copper (C11000) or 60% copper (C28000) alloy surfaces. *B. subtilis* strains BR151 and SL10950 were grown, harvested, and exposed to the indicated alloy coupons as described in Figures1 and 2, respectively. At the indicated times, the cells were washed from the metal coupon surface with 100 *μ*L of 0.85% NaCl, total DNA isolated, and the recovered DNA size-separated in a 1% agarose gel. The size markers (M) are the lambda *Hind*III. Upper panels: Strain BR151 (*SpoIIA*) exposed to 99.9% copper alloy C11000 (Panel A) or 304 stainless steel (Panel B). Lower panels: Strain SL10950 (*spoIIAB-ACΔ::neo*) exposed to 99.9% copper alloy C11000 (Panel C) and 60% copper alloy C28000 (Panel D).

Using the same procedures described for strains BR151 and SL10950 in Figures1, 2, respectively, the cell suspensions were exposed to 304 stainless steel or 99.90% copper alloy (C11000) coupons. At the indicated times, DNA was isolated from the cells recovered from the coupon and size-separated by agarose gel. Because the Promega Wizard SV DNA purification kit only allows the isolation of DNA from vegetative cells, the results in Figure4 enabled us to follow the degradation of copper alloy contact-sensitive vegetative cell DNA without the complication of any resistant endospore DNA. In strain BR151, significant DNA fragmentation was observed by 10 min in cells exposed to 99.90% copper alloy (C11000) coupons and DNA was totally degraded by 15 min (Fig.4A). No appearance of DNA fragmentation was observed in BR151 vegetative cells exposed to 304 stainless steel-exposed cells during the 20-min exposure (Fig.4B).

Unfortunately, because killing of strain BR151 was so modest even on the pure copper surface, we were not able to confidently compare killing and DNA degradation at times shorter than 30 min. For this reason, we carried out the study using the sporulation-defective strain SL10950. Figure4C and D compare genomic DNA degradation of SL10950 exposed to 99.9% C11000 and 60% C28000 copper alloy coupons, respectively. On the pure copper coupons, DNA fragmentation was not observed at 10 or even 20 sec of exposure, when over 6 logs of cell death had occurred (see Fig.2A). DNA degradation was complete by 30 sec. Following exposure to the 60% copper alloy (C28000), no fragmentation was observed even at 20 min, the time point at which no survivors could be detected (Fig.3A). Thus, cell death preceded DNA fragmentation, as was observed by Hong et al. (2012) in studies of *E. coli*.

### Microscopic visualization of viability and membrane integrity following exposure to copper alloy C11000 (99.9%) surfaces

We used the Invitrogen BacLight kit to assess membrane integrity. The kit utilizes two DNA and RNA binding stains, SYTO®9 and propidium iodide, which differentially enter bacterial cells. The green-fluorescent stain SYTO®9 passes across intact cell membranes while propidium iodide, a red-fluorescent stain, only enters dead or dying cells with damaged membranes. When both dyes are present within the cell, the propidium iodide interferes with SYTO®9 fluorescence and, thus, dead or dying cells with severely compromised membranes stain red, whereas cells with intact membranes stain green.

**Figure 5:**
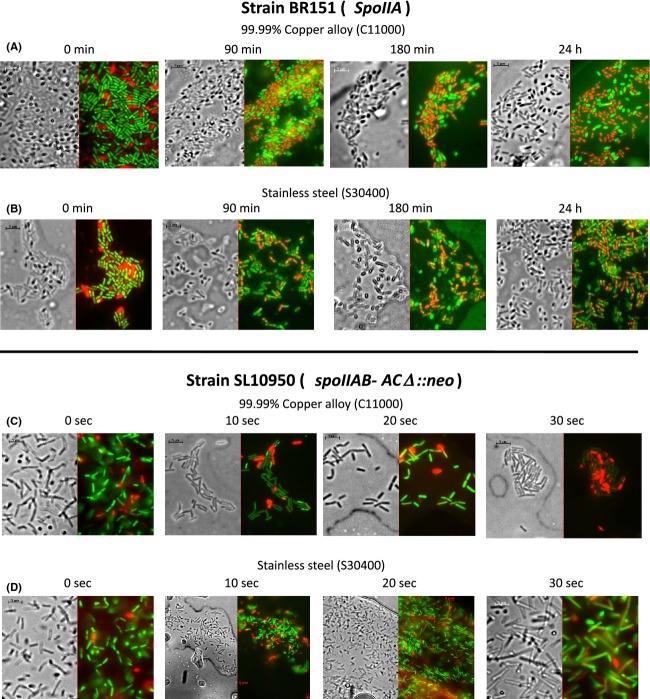
Microscopic assay of membrane integrity following exposure to 99.90% copper surface. *Bacillus subtilis* strains BR151 and SL10950 were grown, harvested, and exposed to 99.90% copper (C11000) or 304 stainless steel (S30400) coupons, as described in Figures1 and 2, respectively. At the indicated time of exposure, the cells were washed from the coupon surface and prepared for the Live/Dead BacLight Assay. Total magnification used was 1000× oil immersion.

We used the BacLight kit to follow the impact of exposure to metal alloy surfaces in *B. subtilis* strains BR151 and SL10950. The strains were grown, harvested, resuspended in 0.85% NaCl, and the cell suspension exposed to 304 stainless steel or 99.90% copper alloy (C11000) coupons as described in Figures1, 2, respectively. At the indicated times, the exposed cells were washed from the coupon and the recovered cells stained for observation by phase and fluorescent microscopy. The results are shown in Figure5.

Strain BR151 did not appear to exhibit a dramatic decrease in the percentage of green-fluorescent cells following exposure to the copper coupons (photo row A) or the 304 stainless steel coupons (photo row B). In contrast, following a 30 sec exposure of the sporulation-defective strain SL10950 to the 99.9% copper alloy surface a preponderance of strongly red-fluorescent cells was observed (photo row C). Interestingly, at the 20-sec time point at which time an approximate 6 logs of killing (see Fig.2A), no significant increase in the percentage of red-fluorescent cells was evident. Hong et al. (2012) obtained similar findings in their studies of *E. coli*. Despite over 5 logs of cell death no significant increase in the number of red-fluorescent cells was observed. Not until 45 min exposure, at which point few if any survivors were detected, could a preponderance of red-fluorescent cells be seen.

## Discussion

The cell envelopes surrounding gram-positive and gram-negative cells are structurally complex and differ significantly. Previous studies of the role of lipid peroxidation in copper alloy surface killing were carried out on the gram-negative *E. coli* (Hong et al. 2012). Thus, because *B. subtilis* is both gram-positive and spore forming, we anticipated differences in its response to copper alloy surface exposure. The inner most envelope layer of gram-positive and gram-negative cells is a typical plasma membrane consisting of a phospholipid bilayer and containing a variety of associated and transmembrane proteins. In addition to this plasma membrane, gram-positive and gram-negative bacteria contain extracellular structural layers with major differences (reviewed in Schuster and Sleytr 2000; Silhavy et al. 2010; Schneewind and Missiakas 2012; Errington 2013). Gram-negative bacteria have a three-layered cell envelope: the plasma membrane or inner membrane (IM), an outer membrane (OM) that is the distinguishing characteristic of the gram-negative bacteria, and a peptidoglycan cell wall that lies between the OM and IM. The OM is also a lipid bilayer but consists of a glycolipid outer leaflet and a phospholipid inner leaflet as well as a number of associated lipoproteins and *β*-barrel transmembrane proteins. Between the inner and outer membranes is an aqueous space called the periplasm that contains its own distinct species of proteins. In contrast, gram-positive bacteria lack an outer membrane and the peptidoglycan layer is significantly thicker than that found in gram-negative bacteria. Variations in each of these extracellular layers further distinguish different strains of gram-negative and gram-positive species. Finally, surrounding all of this in many but not all gram-positive and gram-negative cells is a monomolecular crystalline array of protein subunits called the S-layer (Sára and Sleytr 2000; Claus et al. 2005).

The results reported here demonstrate that *B. subtilis* vegetative cells but not endospores are sensitive to killing by copper alloy surfaces. We observed <1-log of killing in the initial 45 min of exposure of strain B151 (Fig.1A) but the DNA degradation studies reported in Figure4A suggest that the vegetative cells in this mixed culture were actually killed in less than 15 min of exposure and are likely solely responsible for the observed cell death. Weaver et al. (2008) reported copper surface resistance of *Clostridium difficile* endospores, a gram-positive pathogen. They observed rapid killing of better than 2-logs in the first 1 or 2 h of copper alloy exposure, which they propose to be due to the killing of vegetative cells, followed by a slow steady decreased viability over the next 24–48 h resulting from loss of the partially copper-resistant *C. difficile* spores. *B. subtilis* spores appear to be more resistant to attack on copper surfaces since the rate of killing between 45 min and 24–48 h (Fig.1A) is extremely slow with significant survival even after 48 h exposure. Similarly, *B. anthracis* endospores appear to be more resistant to copper surface killing than *C. difficile spores*. Bleichert et al. (2014) reported that exposure of a *B. anthracis* culture containing both vegetative cells and endospores to a pure copper surface caused about a 2-log loss in viability in the first 15 min of exposure but little or no decrease in survival over the following 24 h. They demonstrated that only the vegetative cells were killed by copper surface exposure.

We surmised that, like *C. difficile* and *B. anthracis*, *B. subtilis* spores are copper-contact resistant and tested this hypothesis using a sporulation-defective *B. subtilis* strain SL10950 that carries a nearly complete deletion of the *SpoIIA* operon. Strain SL10950 is surprisingly sensitive to copper alloy surface killing (Figs.2A, 3A). Only a short exposure (less than a minute in the case of alloy C11000) produced over 10 logs of killing. The copper-contact sensitivity of strain SL10950 appears to be even greater than that of strain B151 vegetative cells, in which genomic DNA degradation initiated after about 10 min of copper surface exposure (Fig.4). The reasons for this apparent increased sensitivity have not been determined. The products of the *SpoIIA* operon are not the initial regulators of sporulation, a role played by the products of the *Spo0A* operon (Fawcett et al. 2000; Sonenshein 2000; Molle et al. 2003; Piggot and Hilbert 2004; De Hoon et al. 2010). It is possible that changes in cell envelope structure in the *spoIIAB-ACΔ* strain exacerbate copper-contact sensitivity.

The results shown in Figures1B, 2B, 3B, demonstrated that, as reported by Hong et al. (2012), exposure to copper alloy surfaces stimulates lipid peroxidation and that lipid peroxidation correlates with vegetative cell killing during exposure to copper surfaces in both the Spo^+^ and Spo^−^ strains. Figure3 is particularly informative. Here, by exposing the sporulation-defective strain to a 60% copper alloy (C28000) we were able to slow down both the rate of both killing and lipid peroxidation. Under this condition it is very clear that these processes are tightly correlated and, most notably, both killing and peroxidation initiated immediately and continued at a uniform rate for the first 20 min of the experiment. The peak of peroxidation was reached at 20 min exposure, the same time point at which no significant survivors were detected. Using the same 60% copper alloy (C28000) in studies of *E. coli*, Hong et al. (2012) found a 30-min delay in the onset of lipid peroxidation that correlated with a 30 min delay in cell death and resulted in a biphasic killing curve. We suggest that the differences in the kinetics of killing between these two bacterial species, immediate onset versus biphasic, results from structural differences in the cell envelope between the gram-negative *E. coli* and gram-positive *B. subtilis*. The outer membrane of *E. coli* could provide a partial barrier to the reactive oxygen species formed by the Fenton reaction and copper surface exposure thereby delaying membrane lipid peroxidation and cell death. Studies are underway with a variety of bacterial species to test this hypothesis.

The mechanism of lipid peroxidation has been extensively studied in mammalian cells that contain high levels of polyunsaturated fatty acids and some have questioned whether similar processes can occur in bacteria which are known to contain only monounsaturated fatty acids (reviewed in Imlay 2003; Zhang and Rock 2008; Lemire et al. 2013). The basis of their concerns comes from an in vitro study of the oxidation of unsaturated fatty acids that found that peroxidation occurs only in *cis*-polyunsaturated fatty acids (Bielski et al. 1983). We suggest that the in vitro reactions, which were carried out in ethanolic solutions, do not adequately mimic the in vivo situation of the plasma membrane. Solutions cannot reproduce the close proximity of the fatty acid side chains found in vivo in lipid bilayer membranes of cells. Such close positioning could allow lipid peroxide chain reactions between closely associated monounsaturated fatty acids in a phospholipid leaflet.

Others have suggested that DNA degradation could be the primary target of copper-contact killing (Warnes et al. 2010, 2012; Weaver et al. 2010; Warnes and Keevil 2011) but the results reported here and those of Hong et al. (2012) clearly contradict this proposal. The results in Figure4D demonstrated that, in the *B. subtilis* sporulation-defective strain SL10950 exposed to the 60% copper alloy, cell death occurred prior to the onset of DNA degradation. Hong et al. (2012) obtained similar results in *E. coli*. They compared survival, lipid peroxidation, and genomic DNA degradation on a number of copper alloy surfaces and observed no genomic DNA degradation in *E. coli* cells exposed to the same 60% copper alloy used here despite near complete killing. Thus, in both *E. coli* and *B. subtilis* DNA degradation is a secondary event in copper alloy contact killing.

In summary, the results presented here indicate that *B. subtilis* vegetative cells are sensitive to copper alloy surface killing by mechanisms similar to those we reported in *E. coli* (Hong et al. 2012). That is, the initiating event is the nonenzymatic copper-dependent peroxidation of unsaturated fatty acids in the bacterial membrane. This lipid peroxidation leads to a breakdown of the structural and/or functional integrity of the membrane that, in turn, stimulates the rapid, efficient, and catastrophic cell death observed in bacterial cells exposed to dry metallic copper alloy surfaces. Unsaturated fatty acids are essential and irreplaceable components of biological membranes (Cronan and Gelmann 1975; Fujita et al. 2007; Zhang and Rock 2008; Zhu et al. 2012). Differences in the fatty acid composition of the lipid bilayer impact the membrane's physical properties and thereby indirectly regulate the activity of integral membrane proteins, many of which are required for cell viability. In the case of *E. coli*, the minimal level of membrane unsaturated fatty acids is 15–20% and mutations that block unsaturated fatty acid synthesis or decrease levels to below this threshold minimum are lethal (Cronan and Gelmann 1973). Mutations leading to the loss of the ability to synthesize these targets of copper alloy surface induced peroxidation might provide some level of copper surface resistance but would likely be lethal events. Consistent with this, mutant strains resistant to copper alloy surface-mediated killing have yet to be reported. Those rare survivors found on copper surfaces do not carry heritable alterations that confer the ability to survive copper alloy surface exposure and most likely their survival is the result of undefined sporadic effects (Wilks et al. 2005).
